# Genetic characterization and quantitative trait relationship using multivariate techniques reveal diversity among tomato germplasms

**DOI:** 10.1002/fsn3.2850

**Published:** 2022-04-18

**Authors:** Chikezie Onuora Ene, Wosene Gebreselassie Abtew, Happiness Ogba Oselebe, Friday Ugadu Ozi, Ugochukwu Nathaniel Ikeogu

**Affiliations:** ^1^ Department of Horticulture and Plant Sciences Jimma University Jimma Ethiopia; ^2^ 439477 Department of Agriculture Alex Ekwueme Federal University Ndufu‐Alike Abakaliki Nigeria; ^3^ 125795 Department of Crop Production and Landscape Management Ebonyi State University Abakaliki Nigeria; ^4^ Section of Plant Breeding and Genetics Cornell University Ithaca New York USA

**Keywords:** cluster analysis, fruit yield, GT biplot, principal components, scree plot, tomato

## Abstract

Tomato accessions collected from different sources were evaluated to study their diversity, genotype–traits association, as well as pinpoint most selective trait(s) in a controlled environment in Jimma, Ethiopia. The two terms pot experiments were carried out in randomized complete block design with three replications. The genotype–trait (GT) biplot revealed high percentage variability above 70% in related growth traits for the first and second principal components (PC) summed up, in the two trials, whereas related floral and fruit traits association indicated medium to high (55%–65%) total explained variations in both seasons. It further showed that ‘wild parent’, ‘CLN2498D’, ‘CLN2498F’, ‘UC Dan India’, ‘Ruma’, ‘PT4722A’, ‘CLN2679F’, ‘CLN2585C’ and ‘CLN2585D’ were the best performers in most of the related growth, floral, and fruit traits in those seasons. Principal component analysis showed that traits, such as plant height, number of branches, leaves, nodes, internodes, stem girth, style length, stigma length and diameter, flower length and width, number of flowers per truss, number of fruits per truss, and fruit weight per plant, in the first dimension were positively related to yield and consistent with high loading factors in both seasons and could be underpinned highly important in breeding for increased fruit yield. Clustering and its comparison of means showed that ‘CLN2498D’, ‘PT4722A’, ‘Ruma’, ‘Tropimech’, and ‘UC Dan India’ of cluster I in both trials expressed the best traits including related growth, floral, and fruit traits. Therefore, selection for any trait would favor accessions in this cluster.

## Graphical abstract

GT biplot projected ‘wild parent’, ‘CLN2498D’, and ‘UC Dan India’ as best performers for most of the related growth, floral and fruit traits in both seasons. PCA in both seasons revealed that selection for most related growth, floral and fruit traits such as leaf area, number of branches, number of flowers per truss, flower length, number of fruits per truss, fruit weight per plant, and total fruit yield increased tomato fruit yield. Cluster analysis revealed high genetic variation among accessions.

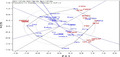

## 1 INTRODUCTION

Tomato (*Solanum lycopersicum* L.) is one of the most relevant productions of the agro‐horticultural sector in not just Ethiopia but also around the world (El‐Mansy et al., 2021; Tolessa et al., 2013; Worku & Sahe, 2018) for its edible fruits. This nutritious fruit is a valuable vegetable which could be taken raw as salad, processed as puree, ketchup, juice, or powder, and cooked as tomato sauce or soup which has been reported to be an effective remediation for sufferers of constipation (Alda et al., 2009; Rehman et al., 2019). Tomato supplies phytochemicals such as: β‐carotene, flavonoids, lycopene, vitamins, and vital minerals, which to a great degree contribute to keep deficiency diseases away from man coupled with its cash‐generating ability for smallholders and medium‐scale commercial farmers, being a relatively short‐duration and high‐yielding crop (Martí et al., 2018).

The increase in demands of fresh tomato fruits and their products has necessitated the quest for high yielding varieties among tomato farmers. However, the high yielding cultivars are insufficient to meet up with the fast‐growing global fresh fruit demands (Asfaw, 2021). The biotic and abiotic factors such as disease incidence and high humidity associated with most tomato production environments are believed to contribute greatly to the low production (Atugwu et al., 2019). Also, the few available cultivars are out of farmers’ reach possibly because of high cost of high premium varietal seeds especially hybrids, alongside other factors. These could possibly be responsible for the low annual fruit yield in Ethiopia and most surrounding East African countries (Tolessa et al., 2013). Hence, there is a need for an extensive germplasm recollection including the wild relatives, characterization, selection, and exploitation of selected but unknown genetic materials. This would help to improve tomato resistance to diseases, adaptability to humid environments, and enhance fruit yield and quality for present and future gain. Evaluation would be helpful in understanding the breeding values and genetic background of the available materials. This would definitely increase tomato productivity in Ethiopia and environs. Ng (1991) earlier opined that genetic resources can only be useful to plant breeders and other plant users when they have been known through adequate characterization and evaluation. By this, breeders can investigate diversity of the species involved to consider perhaps direct introduction as cultivars, or provide useful variation in a breeding program.

Germplasm evaluation is always confronted by two major challenges. The first is genotype‐by‐environment interaction (GEI) for a particular trait, and the second is unfavorable correlation among influencing traits as well as trait relations of genotypes (Yan & Frégeau‐Reid, 2018). El‐Aziz et al. (2016) reported that selection of tomato genotypes with superior performance in different spatial environments has been studied, but consistency in performance on certain quantitative traits under temporal environment has not received extensive outlook.

Identification of the major multiplex traits which contribute more to higher yield in any crop species during germplasm evaluation is a fundamental objective of any breeding program. Several morphological traits affect tomato fruit yield either positively or negatively. Through a careful examination of the contribution of these component traits, it is easier to concentrate efforts on the traits with higher influence on the primary trait in the future selection process. Through genotype–trait (GT) biplot which applies genotype and genotype‐by‐environment interaction (GGE) biplot technique as proposed by Yan and Rajcan (2002), trait association, and genotype–trait(s)‐specific relations have been visualized graphically. GT biplot has helped breeders to investigate data of various traits at once and this can seriously improve indirect selection of parental lines, unlike most univariate tools which explore traits in a data set separately.

The application of GGE biplot to study GT correlation matrix has been witnessed in some crops species such as soybean (Yan & Rajcan, 2002), buckwheat (Joshi, 2012; Joshi & Okuno, 2010), linseed (Soto‐Cerda et al., 2014), oats (Martin et al., 2014), tartary sunflower (De C. Leite & de Oliveira, 2015), forage sorghum (Aruna et al., 2016), Ethiopian white lupin (Atnaf et al., 2017), durum wheat (Kendal, 2019; Mohammadi, 2019), Sesame (Boureima & Yaou, 2019), and maize (Munawar et al., 2013; Shojaei et al., 2020). Although many reports have implicated the application of GT analysis to discover superior accessions in many crops species, there is paucity of information on relationship between genotypes and the quantitative traits of related growth, floral, and fruit simultaneously in tomato germplasm especially in a controlled environment. Recently, the GT biplot technique was used to assess the adaptability of advanced generations of wild and cultivated tomato crosses under open field condition (Atugwu et al., 2019), and greenhouse tomato germplasm characterization for NaCl tolerance only at the seedling stage **(**Rehman et al., 2019).

Cluster analysis has often been used recently as a genetic tool to give spotlights on the quality of relatedness of the genetic materials based on the traits under consideration. It separates the accessions into dissimilar groups based on Euclidian distance (Subramanian & Subbaraman, 2010) for easy selection. Principal component analysis (PCA) shows the amount of contributions of the traits – whether so much, a few, or zero contribution – to the observed variation witnessed among accessions. It suggests which trait expresses higher variability based on its magnitude and qualifies such trait(s) as the most selective among accessions (Ene et al., 2016b). In the GT biplot, the first two principal components (Dimension 1 – primary effects; and Dimension 2 – secondary effects) from the data are plotted. If they cannot provide complete percentage of explained variances in the data, other dimensions may be X‐rayed using scree plot or related output (Aruna et al., 2016).

In the present investigation, GT biplot was used to select the tomato accessions using multiple‐trait data. The cultivated tomato germplasm including a wild parent of *Solanum pimpinellifolium* L. species collected from different agro‐ecological sources was screened together for two seasons under a controlled environment. The main objectives of the study were (1) to evaluate tomato accessions using cluster analysis and PCA as genetic tools to check relatedness among accessions and most discriminating trait(s), respectively; (2) to understand trait associations in tomato germplasm using GT biplot analysis; and (3) to identify high‐ and low‐performing accessions in the studied traits that could warrant selection for the development of interspecific breeding/mapping populations, which could possibly go in for a QTL linkage mapping.

## 2 MATERIALS AND METHODS

### 2.1 Plant material, site description, and layout

Seed samples of different sources including: a landrace, accessions and cultivars totaling 35, namely ‘CLN2116B’, ‘CLN2468B’, ‘CLN2498D’, ‘CLN2498F’, ‘CLN2545B’, ‘CLN2585C’, ‘CLN2585D’, ‘CLN2679F’, ‘CLN2714G’, ‘CLN2714H’, ‘CLN2762A’, ‘CLN2768A’, ‘CLN2777A’, ‘CLN2777E’, ‘CLN2777F’, ‘CLN2777G’, ‘CLN2777H’, ‘PT4722A’, and ‘PT4722B’ (Melkassa Agricultural Research Center, Ethiopia), ‘Dan Holland’, ‘Darika’, ‘Ruma’, ‘Rukuta’, ‘UC 82B’, ‘UCT’, and ‘UC Dan India’ (Nigerian local farmers), ‘Gadar’, ‘Rio Grande’, and ‘Roma Savanna’ (National Horticultural Research Institute, Ibadan‐Nigeria), ‘Tima’, ‘Tropimech’, and ‘Roma VF‐Nig’ (Market, Nigeria), ‘Roma VF‐Ethio’ and ‘Melka Salsa’ (Ethiopian local farmers landrace), and ‘Wild parent’ of *S. pimpinellifolium* (University of Nigeria, Nsukka, Nigeria) were collected in Nigeria and Ethiopia alike. All the plant materials expressed determinate growth habits except the ‘wild parent’ of *S. pimpinellifolium,* which showed indeterminate growth habit. The seeds were evaluated for related growth, floral, and fruit traits for two seasons in the greenhouse of Department of Horticulture and Plant Sciences, Jimma University, Ethiopia, from July to November 2019 (first season) and January to May 2020 (second season), respectively. Jimma has its bearing on latitude 7^o^4″N and longitude 36^o^50″E with the altitude of 1,710 m above sea level and is located in southwest Ethiopia. The mean minimum and maximum temperature are about 11.4°C and 28°C, respectively, while the average annual rainfall is a little above 1,500 mm which occurs from April to October. The relative humidity is 37.92% and 94.4% as minimum and maximum, respectively (BPEDORS, 2000).

Seeds were sowed in plastic trays filled with a mixture of carbonated rice husks and a bit of topsoil to raise the seedlings of all the accessions in the greenhouse. The nursery routine practices were observed which aided the production of vibrant seedlings that were transplanted on the 26th day after germination into the 28cm experimental pots well arranged in fixed position laid out in a three replicate randomized complete block design under a greenhouse environmental condition. The pots were filled with well‐mixed organically enriched compost together with topsoil based on the required standard, as recommended by Agong et al. (1997).

### 2.2 Agronomic practices

All the standard horticultural practices required for a greenhouse tomato production such as irrigation, weed picking, fungicide (Ridomil‐Mancozeb and Metalaxyl‐M), insecticide (Karate‐Lambda‐Cyhalothrin 5% EC), and fertilizer (DAP‐Di ammonium Phosphate), as recommended in the production labels to curtail fungi and insect attack and maintain healthy growth, were applied. Observations were carried out and records were taken on five randomly selected plants per accession for each replicate.

### 2.3 Recorded observations

Data were recorded on the following related growth, floral, and fruit traits.

#### 2.3.1 Related growth traits

These traits were recorded at 9 weeks after transplanting. Plant height (cm) was taken using meter tape from the plant base to the shoot tip, leaf length (cm) was taken using meter tape from the point of attachment to the petiole to the leaf tip, and leaf width (cm) was taken using meter tape at the widest point of the leaf. Leaf area (cm^2^) was calculated using the formula, *X* = 0.5 × *L* × *W*, according to Carmassi et al. (2007a) (where *X* = leaf area, 0.5=constant, L=leaflength, and W=leafwidth), number of leaves, number of branches, number of nodes, and number of internodes were all counted and stem girth (cm) measured by vernier caliper.

#### 2.3.2 Related floral traits

Related floral traits include number of days to first anthesis, number of days to 50% anthesis, number of flowers per truss, total number of flowers per plant, number of aborted flowers per plant, flower length (cm), and flower width (cm). Others included: style length, style diameter, stigma length, and stigma diameter, all in centimeter using a moticam with Motic Image Plus 2.0 software. Ovary length and ovary diameter were taken using ocular micrometer, also in centimeter. This was done after harvesting a given flower from sampled plants and taken to laboratory and cut longitudinally to expose the ovary and other floral parts mentioned. The ovary area (cm^2^) and ovary perimeter (cm) were calculated uniformly using the formula for area (*πr* ^2^) and circumference (2*πr*) of a circle, respectively, it being circular in shape according to Nnungu and Uguru (2014). Where $\pi =\frac{22}{7}$, *r* = radius which is half of the ovary diameter.

#### 2.3.3 Related fruit traits

These data included: number of days to first fruit emergence, number of days to 50% fruit set, number of days to first fruit ripening, number of days to first fruit spoilage, number of days to 50% fruit spoilage, and number of days to 100% fruit spoilage. From each accession, eight fruits were randomly picked after the second harvest to measure fruit length (cm) and diameter (cm) using vernier calipers. Fruits were cut crosswise to measure pericarp thickness (cm) using vernier calipers. The number of locules per fruit was counted at the same time. Number of fruits per truss and total number of fruits per plant were counted. The total number of mature fruits showing ripening initiation at second harvest was weighed with electronic weighing balance and the fruit weight per plant recorded. Average fruit weight was obtained by dividing fruit weight per plant by total number of fruits per plant while the total fruit yield per hectare was estimated from the fruit weight.

### 2.4 Data statistical analysis

All statistical analysis was done separately for each evaluation season.

#### 2.4.1 Genotype‐by‐trait biplot analysis

The collected data of the abovementioned quantitative traits were subjected to ANOVA as described by Steel et al. (1997) to obtain significant genotypic differences. Data were further analyzed by the multivariate technique ‘genotype‐by‐trait biplots’, an option of GGE biplot software version 6.3 (Yan, 2001) on separate data from each season using ‘Scaling 1’. For phenotypic correlations among traits according to Yan and Tinker (2005), trait‐focused singular value partitioning (SVP = 2) was employed while a tester‐centered (centering 2) GGE biplot was generated. Here, traits were regarded as ‘tester’ when using ‘relation among testers’ option. The. ‘which‐won‐where’ polygon option was used to identify which accession was the best in a given set of traits, and hence, identify the super accession(s).

#### 2.4.2 Principal component and hierarchical cluster analyses

Principal component analysis (PCA) and hierarchical cluster analysis following Ward's method were done on the data for the two seasons separately. PCA was to show the contribution of each trait to the total phenotypic variations observed among the tomato accessions, that is, to indicate the trait(s) with the highest selective ability based on the magnitude of loadings, while cluster Euclidean distance showed level of association among accessions. The two analyses were done using R statistical software version 3.2.0 (Kabacoff, 2012) (http://www.r‐project.org).

## 3 RESULTS AND DISCUSSION

### 3.1 Trait associations through genotype‐by‐trait biplot

**FIGURE 1&2:**
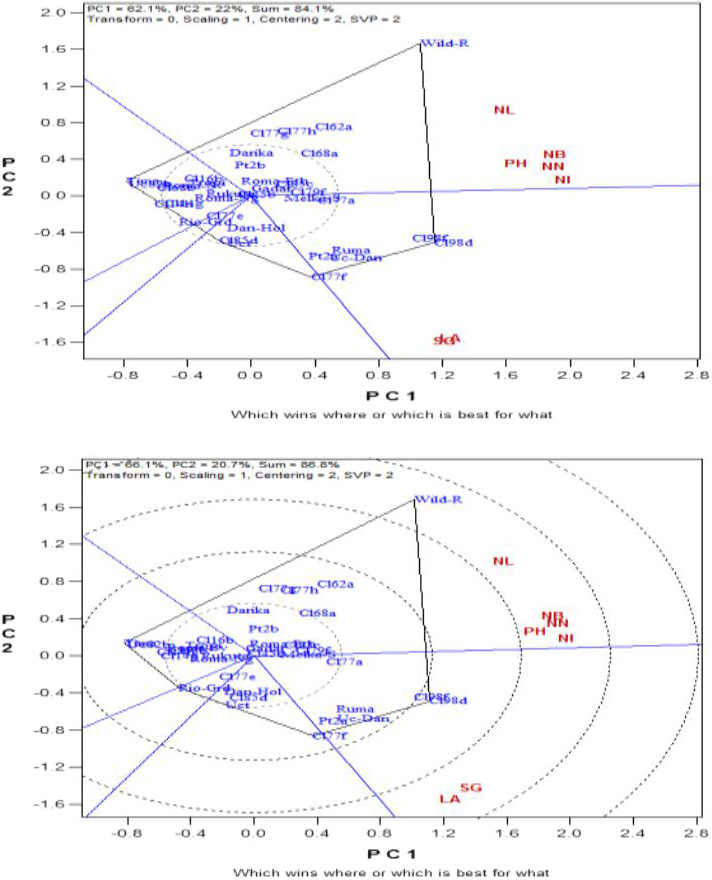
Biplot showing the growth traits across 35 genotypes during the first and second evaluation trials

The which‐won‐where view of the GT biplot was used for the study under traits association through genotype‐by‐trait biplot analysis. The ‘which is best for what’ view of the GT biplot showing the visualization of the relationships among the related growth traits across tomato accessions during the first and second evaluation trials is presented in Figures 1 and 2, respectively. This analysis was to help make a comparison among tomato accessions on the basis of multiple related growth traits numbering 7 and to identify superior accession(s) for the given trait(s). The total variations explained by the PC1 and PC2 for the related growth traits in the first evaluation biplot were 84.1% while the second planting showed 86.8%. The first evaluation trial (Figure 1) showed that the ‘wild parent’ had high performance for number of leaves, number of branches, plant height, number of nodes, and number of internodes. ‘CLN2498D’ and ‘CLN2498F’ were better for leaf area and stem girth followed by ‘CLN2777F’, ‘Ruma’, ‘PT4722A’, and ‘UC Dan India’ in no particular order but based on proximity in the sector. The rest accessions showed very low performance for the entire traits. Similar result was also found in the second GT biplot evaluation trial (Figure 2).

**FIGURE 3&4:**
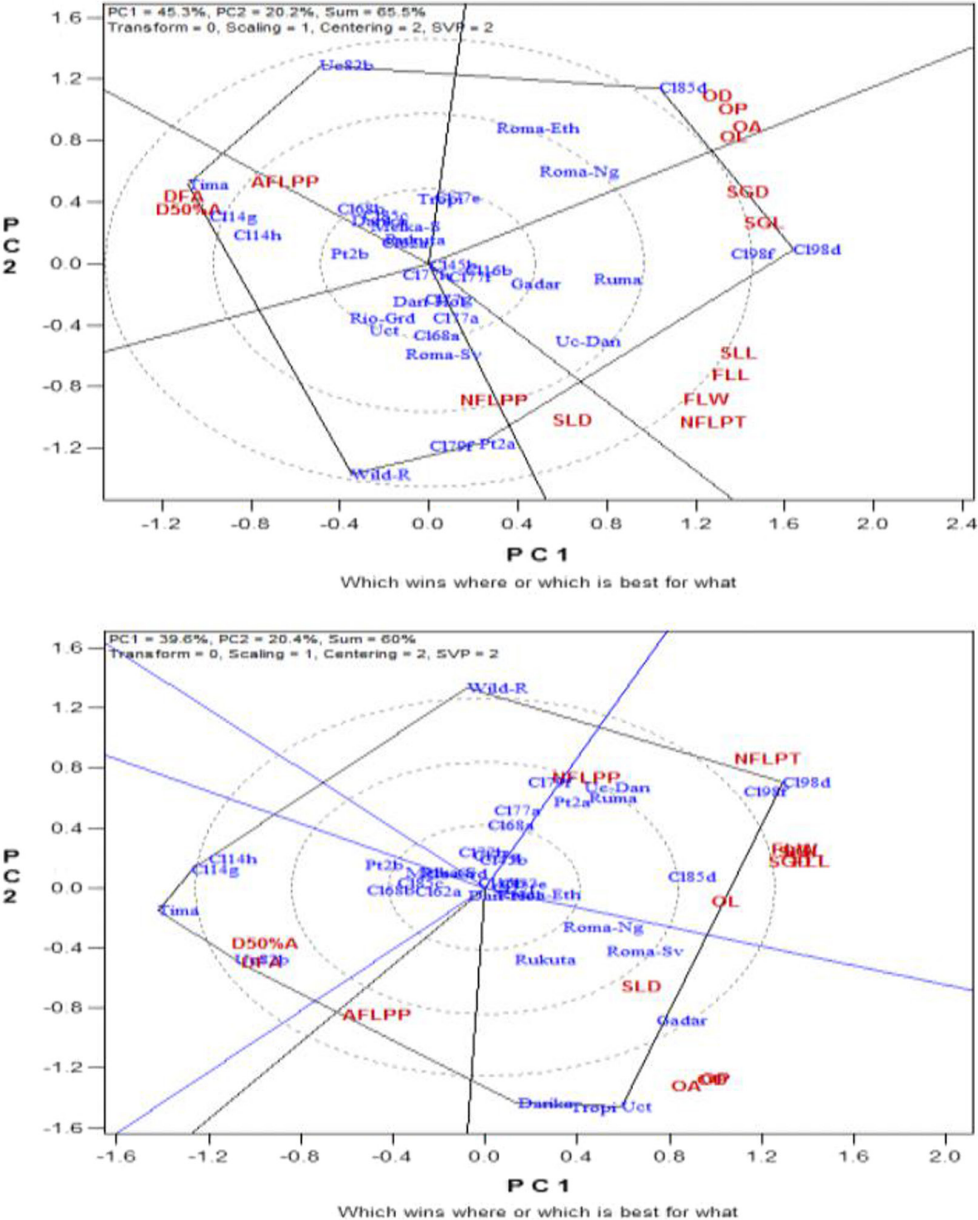
Biplot showing the floral traits across 35 genotypes during the first and second evaluation trials

Figures 3 and 4 show the ‘which won where’ image of the GT biplot, which projects the correlation between the related floral traits and 35 tomato accessions in the course of the first and second trials, respectively. ‘CLN2585D’ was best performer for ovary area, ovary length, ovary diameter, and ovary perimeter as expressed in the first evaluation (Figure 3). ‘CLN2498D’ and ‘CLN2498F’ were the best performer for stigma length and stigma diameter, which also showed good performance in style length, flower length, and flower width as well as number of flowers per truss. ‘Wild parent’ was ranked best for number of flowers per plant, whereas ‘PT4722A’ and ‘CLN2679F’ showed the leading performance in style diameter. ‘Tima’ took the longest number of days to express flower anthesis as well as 50% anthesis followed by ‘CLN2714G’ and ‘CLN2714H’. ‘UC 82 B’ recorded highest number of aborted flowers per plant followed by ‘Tima’. The rest accessions showed very low performance for the entire related floral traits. The accessions that clustered around the point where the radiate lines meet, which is the center of the polygon, showed average performance for the entire characters measured. The total variance accounted for by the PC1 and PC2 in the first experiment was 65.5% while 60% was explained in the second trial. For the second experiment for related floral traits association (Figure 4), ‘CLN2498D’ and ‘CLN2498F’ maintained similar but high‐performance trend for most traits as in the first trial, alongside ‘CLN2585D’ which was champion for ovary length. ‘Wild parent’ showed the highest total number of flowers per plant, followed by ‘CLN2498D’, ‘CLN2498F’, ‘UC Dan India’, ‘Ruma’, ‘PT4722A’, and ‘CLN2679F’ that shared average performance for similar trait. ‘Darika’, ‘Tropimech’, ‘UCT’, and ‘Gadar’ showed the best performance for ovary area, ovary diameter, and ovary perimeter as well as style diameter. ‘Tima’ and ‘UC 82 B’ shared low‐performing attribute in that they took longer number of days to observe first anthesis, 50% anthesis, and aborted the highest number of flowers per plant.

**FIGURE 5&6:**
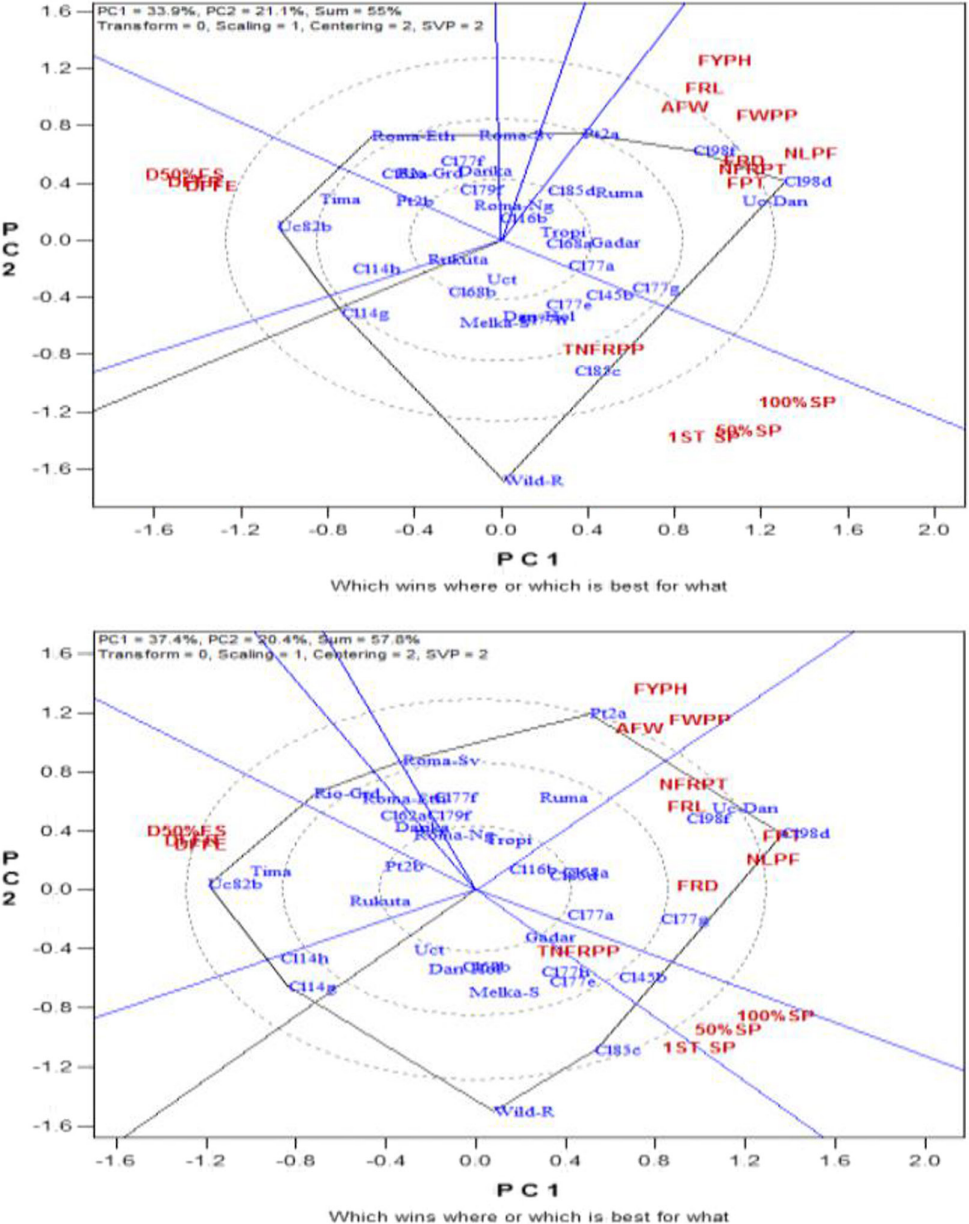
Biplots showing the fruit traits across 35 genotypes during the first and second evaluation trials

The biplot analyses of the tomato accessions by related fruit traits association performance for the two term plantings are shown in Figures 5 and 6. The total variations explained by the PC1 and PC2 in the first and second experiments are represented as 55% and 57.8%, respectively. In the first season trial, ‘CLN2498D’, ‘CLN2498F’, and ‘UC Dan India’ showed good performance for most of the yield‐contributing characters such as: fruit length, fruit weight per plant, average fruit weight, fruit diameter, number of locules per fruit, fruit pericarp thickness, number of fruits per truss, and fruit yields per hectare (Figure 5). ‘Wild parent’ maintained the same high‐performance trend as in most of the related growth traits but this time for related fruit traits such as total number of fruits per plant, whereas ‘wild parent’ together with ‘CLN2585C’ demonstrated longer number of days to first, 50%, and 100% fruit spoilage. The highest number of days to attain first fruit emergence, 50% fruit set, and first fruit ripening was recorded in ‘UC 82 B’, ‘Tima’, ‘CLN2714H’, and ‘CLN2714G’. Similar result was also obtained just like in the first trial except that in the second trial; ‘PT4722A’ ranked the best for average fruit weight, fruit weight per plant, and fruit yield per hectare. ‘UC Dan India’, ‘CLN2498D’, and ‘CLN2498F’ took the lead for number of fruits per truss, fruit length, fruit diameter, fruit pericarp thickness, and number of locules per fruit (Figure 6). From the biplots of the first and second trials, no tomato accession displayed clear‐cut average performance for all the related fruit traits studied especially in the second evaluation trial. The rest accessions only showed low performance for the entire traits. For all traits under consideration in both trials, the total variations explained by the PC1 and PC2 combined ranged from 55% for related fruit traits association in the first experiment to 86.8% in the second season biplot for related growth traits.

The GT biplot analysis indicated high percentage variability above 70% in related growth traits correlation for PC1 and PC2 put together in the two season trials, whereas GT biplot for both related floral and fruit traits association showed medium‐to‐high (55%–65%) total explained variations in both seasons. This suggests high and medium‐to‐high variability in the performance of the tomato accessions for the related growth and related floral–fruit traits, respectively. According to Yan and Rajcan (2002), the GT biplot analysis is a standard statistical tool that helps breeders to visualize the relationships that exist among traits, and characterize accessions based on variability that exists on multiplex traits. This is in order to identify those accessions that were superperforming in particular trait(s). For the related growth traits relationship in both seasons, ‘wild parent’ indicating promising performance for number of leaves, number of branches, plant height, number of nodes, and number of internodes could be attributed to the differences in genetic make‐up of the accession. This projected ‘wild parent’ as being better than the rest of the accessions for the traits mentioned. The same reason could be attributed to ‘CLN2498D’ and ‘CLN2498F’, which had broader leaves and larger stem girth, believed to be a result of their resistance to both biotic and abiotic stresses in the experimental environment. Similar implications could be likened to those best performing accessions for the related floral and fruit traits for the two seasons. For instance, ‘CLN2585D’ showed excellent performance for ovary diameter, ovary length, ovary perimeter, and ovary area; ‘CLN2498D’ and ‘CLN2498F’ for stigma diameter, stigma length, style length, flower length, flower width, and number of flowers per truss; ‘PT4722A’ and ‘CLN2679F’ for style diameter; and ‘Darika’, ‘Tropimech’, ‘UCT’, and ‘Gadar’ for ovary area, ovary diameter, ovary perimeter, and style diameter. Also, ‘CLN2498D’, ‘CLN2498F’, ‘PT4722A’, and ‘UC Dan India’ had best performance for fruit length, fruit weight per plant, average fruit weight, fruit diameter, number of locules per fruit, fruit pericarp thickness, number of fruits per truss, and fruit yields per hectare; ‘wild parent’ and ‘CLN2585C’ for total number of fruits per plant, and longer number of days to first, 50%, and 100% fruit spoilage. This suggests that these accessions mentioned had a good character combination associated with tomato fruit yield, which could be important or excellent genetic variation sources in any breeding program aimed at improvement of fruit yield. The findings from the current study gave evidence that GT biplot is a perfect tool of analysis for visualizing genotype‐by‐trait statistical data. It could be observed that the tool efficaciously showed the associations among the tomato quantitative traits studied. Also, it allowed for visual and easy comparability among tomato accessions evaluated based on the multiplex traits. Chen and Foolad (1999) and Pratta et al. (2011) disclosed that wild parents especially those of *S*. *pimpinellifolium* have good agronomic traits performance under varying growth conditions and have been involved in many tomato improvement programs as gene source for resistance to both biotic and abiotic stresses. Atugwu et al. (2019) reported excellent performance of the ‘wild parent’ of *S*. *pimpinellifolium* as well as the crosses involving the ‘wild parent’ in most of the quantitative traits studied. Other authors have reported higher performance of some cultivated tomato accessions over others for some quantitative traits as well. For example, Rai et al. (2017) identified 5 tomato genotypes as being formidable parents for future breeding program of the 56 genotypes evaluated. This was because of their higher performances for the number of fruits per cluster, number of fruits per plant, intermodal distance, average fruit weight, yield per plant, plant height, pericarp and locular wall thickness, and harvest duration which are yield improvement traits. Al‐Otayk (2010) had earlier noted that any crop accession(s) that maintained stability and higher yield comparatively across different environments, which may be temporal or spatial, would more likely be selected as a donor parent for planned hybridization or further improvement exercise. ‘Tima’, ‘UC 82 B’, ‘CLN2714G’, and ‘CLN2714H’ taking longer number of days to achieve first flower anthesis, 50% anthesis, as well as having higher number of aborted flowers per plant implies that they were late and poor producers among the accessions studied. This suggests that the late activation of the reproductive phase could possibly have been the reason for the late translocation of the photosynthates to the sink (fruit) thereby decreasing the fruit yield in those accessions. Sonia et al. (2014) and Ene et al. (2016a) in their various works reported that early activation of the reproductive phase was implemental in translocation of the photosynthates to the sink (fruit), which caused an increase in the fruit yield of both tomato and cucumber, respectively. Breeding for earliness in reproductive process including harvest time is no doubt one of the cardinal objectives in plant breeding. These same accessions also consistently maintained poor performance in most of the agronomic traits examined including total fruit yield indicating poor genetic make‐up.

### 3.2 Principal component analysis

The involvement of PCA was to find out to a greater extent more dependable background knowledge of the quantitative traits which would help to determine and classify accessions into groups based on the contribution or performance of the desirable traits for genetic improvement. This tool of analysis tries to determine which trait(s) promoted the highest phenotypic variation or discrimination among crop accessions. Ahmadizadeh and Felenji (2011) reported PCA practicable usage in choosing parental lines for hybridization or breeding intention. Rencher (2002) earlier opined that principal component tool of analysis has always been included in genetic study in order to monitor the mutual relatedness among traits. PCA has also been described as a vital multivariate tool capable of determining genotype genetic divergence by investigating traits interrelationship (Abdi & Williams, 2010). PCA was used to separate the individual contribution of each trait to the total phenotypic variation noticed among tomato accessions.

**FIGURE 7:**
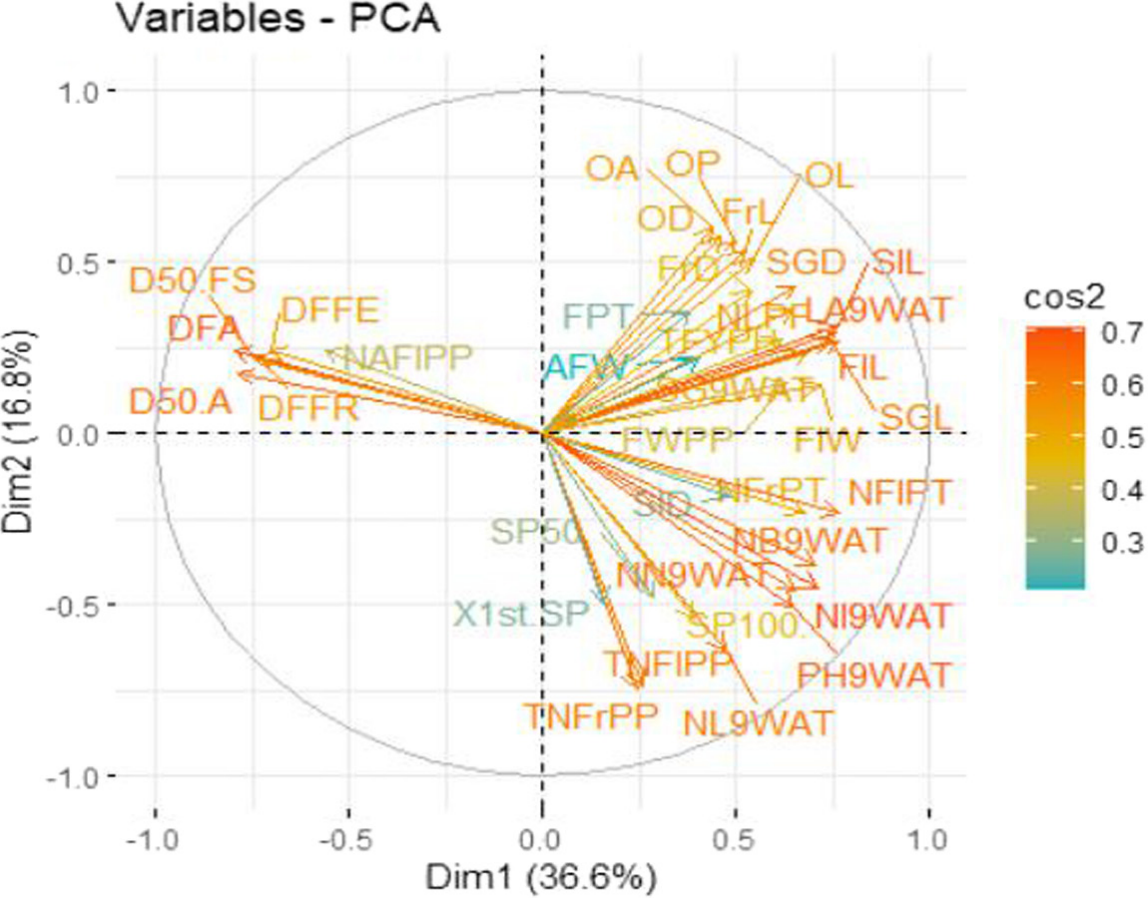
Principal component biplot for some Quantitative Traits of tomato in the 1st season trial

**FIGURE 8:**
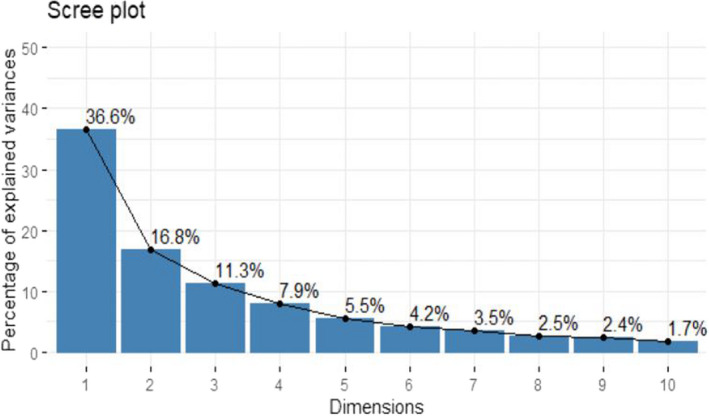
Scree plot for 10 principal components for some quantitative traits in 35 tomato accessions for 1st trial

**FIGURE 9:**
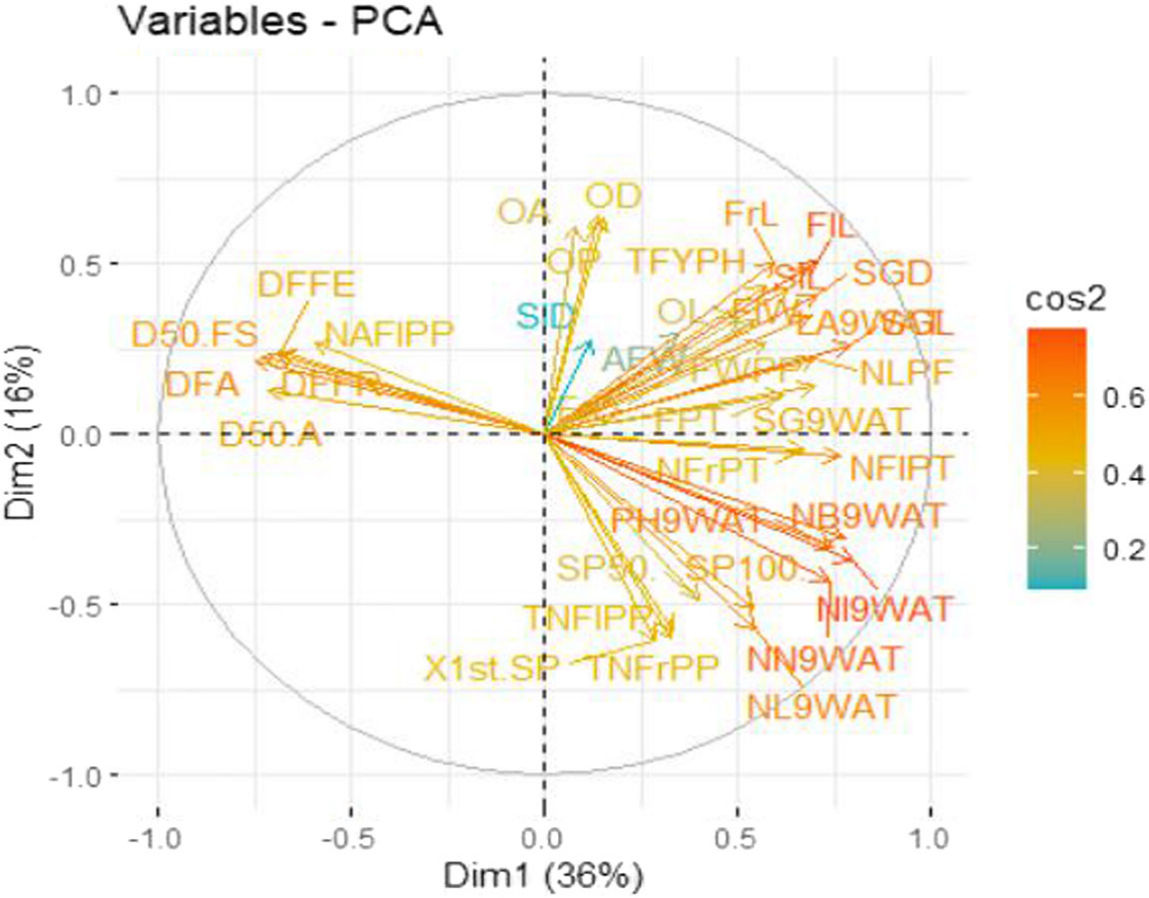
Principal component biplot for some quantitative traits of tomato in the 2nd season trial

**FIGURE 10:**
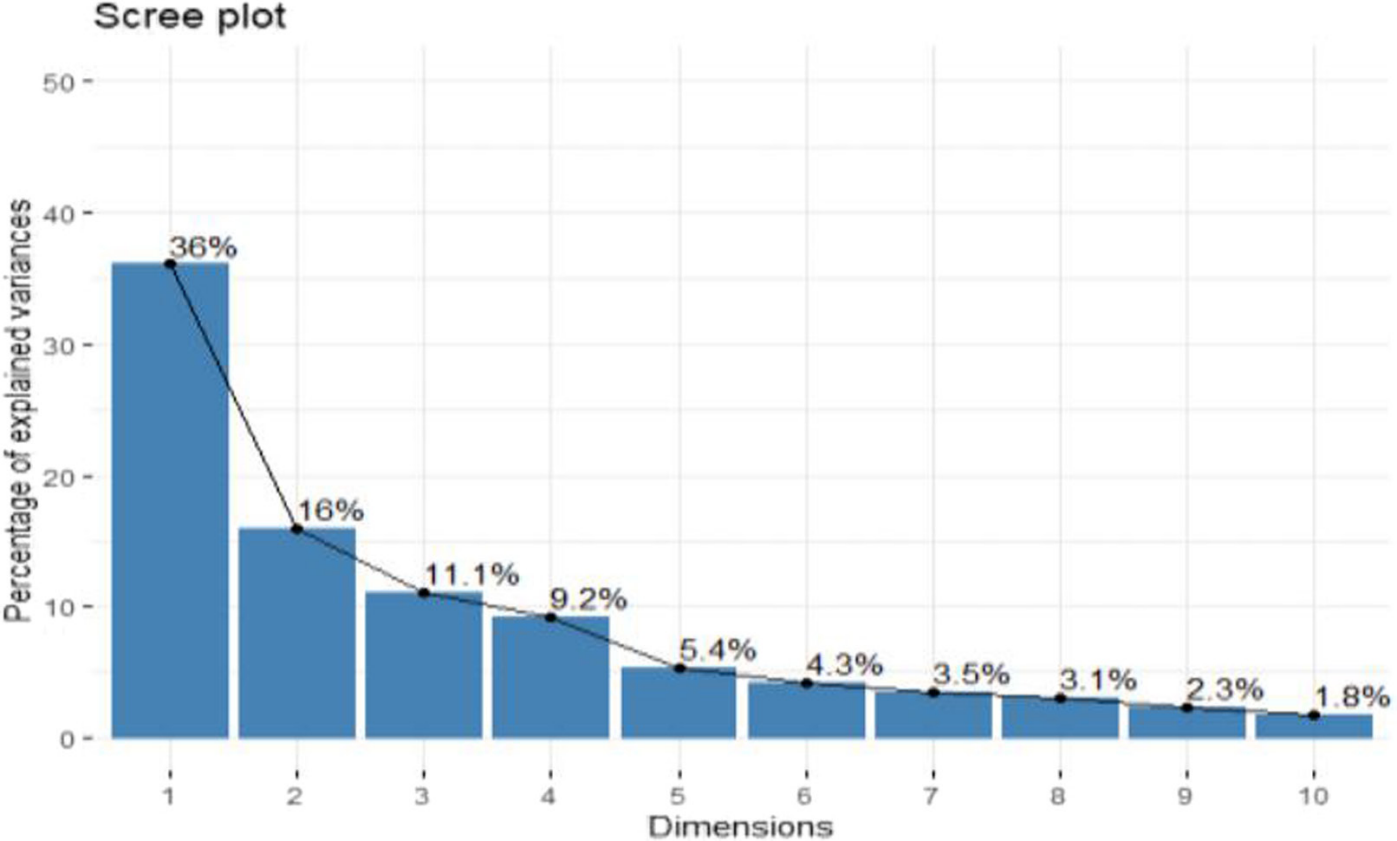
Scree plot for 10 principal components for some quantitative traits in 35 tomato accessions for 2nd trial

Table 1 presents result on principal component analysis based on traits association. The result showed that 78.08% and 77.70% of the total cumulative variability present among the 35 accessions of tomato in the first and second season evaluations, respectively, was explained by the first five principal components with Eigen values of up to 2.0 and above. In the initial trial, principal component 1 (Dim 1), with Eigen value of 13.545, contributed to 36.61% of the total variability, while Dim 2, Dim 3, Dim 4, and Dim 5 with Eigen values of 6.23, 4.17, 2.92, and 2.02 accounted for 16.84%, 11.26%, 7.90%, and 5.47% of total variability, respectively. In the second experiment, 36.02% of the explained variation was recorded at Eigen value of 13.33, whereas the following Eigen values of 5.90, 4.11, 3.41, and 2.01 contributed to 15.96%, 11.09%, 9.20%, and 5.42% of total variance, respectively. The contribution of Dim 1 for variability study among the accessions in both the first (36.61%) and second (36.02%) screening exercise was higher than the other dimensions (Figures 7, 8, 9, 10 and Table 1). The Dim 1 indicated positive factor loadings for all traits in both the first and second planting seasons, except for number of days to flower anthesis, days to 50% anthesis, number of aborted flowers per plant, number of days to first fruit emergence, days to 50% fruit set, and days to first fruit ripening. Dim 2 showed positive factor loading for all traits in both seasons with the exception of plant height, number of leaves, number of branches, number of nodes, number of internodes, number of flowers per truss, total number of flowers per plant, number of fruits per truss, total number of fruits per plant, and number of days to first, 50%, and 100% fruit spoilage. However, style diameter had negative value in the first season but positive in the second, for the same Dim 2. In the first season planting, the majority of factor loadings recorded in Dims 3–5 showed that comparatively Dim 1 represented higher magnitude of positive association with all the quantitative traits studied except average fruit weight recorded in Dim 3, total number of flowers per plant, ovary area, total number of fruits per plant in Dim 4, and number of days to first and 50% fruit spoilage, which were recorded in Dim 5. The results for the second evaluation showed that out of the positive factor loadings recorded in Dims 3–5 for the traits, Dim 1 outshined in magnitude as well indicating higher positive relationship with all the traits examined with the exception of total number of flowers per plant, total number of fruits per plant, average fruit weight recorded in Dim 3, as well as total number of flowers per plant, style diameter, ovary diameter, area, and perimeter, and total number of fruits per plant captured in Dim 4. Also, in the first planting season, the first dimension (Dim 1) was found to be positively and highly associated with number of flowers per truss (0.766), followed by style length (0.761), flower length (0.760), leaf area (0.759), stigma length (0.742), flower width (0.714), number of internodes (0.709), number of branches (0.702), stem girth (0.682), number of fruits per truss (0.674), stigma diameter (0.653), number of nodes (0.652), plant height (0.647), number of locules per fruit (0.643), fruit weight per plant (0.628), total fruit yield per hectare (0.617), etc. in that order. This component has a representation of all the quantitative traits studied, which included related growth, floral, and fruit traits. The second dimension was positively associated with ovary area (0.598), followed by ovary diameter (0.570), ovary perimeter (0.562), fruit length (0.531), ovary length (0.507), stigma diameter (0.429), fruit diameter (0.413), etc. The results for the second season followed slightly a different trend. The first dimension was found to be positively and highly associated with the number of internodes (0.796), accompanied by stigma length (0.792), number of branches (0.774), number of flowers per truss (0.764), plant height (0.744), number of nodes (0.734), flower length (0.705), stigma diameter (0.702), number of locules per fruit (0.699), stem girth (0.698), leaf area (0.687), style length (0.680), number of fruits per truss (0.671), flower width (0.627), and fruit pericarp thickness (0.627) in that order. The Dim 2 was positively related to ovary diameter (0.638), followed by ovary perimeter (0.634), ovary area (0.604), flower length (0.508), fruit length (0.502), style length (0.486), total fruit yield per hectare (0.432), flower width (0.424), stigma diameter (0.407), etc.

**TABLE 1:** Eigen vectors and total percentage variation in quantitative traits among 35 tomato accessions

Traits	First season loadings					Second season loadings				
	Dim. 1	Dim. 2	Dim. 3	Dim. 4	Dim. 5	Dim. 1	Dim. 2	Dim. 3	Dim. 4	Dim. 5
PH9WAT	0.647	−0.508	0.151	0.295	0.189	0.744	−0.336	0.313	0.320	0.056
NL9WAT	0.471	−0.633	−0.033	0.401	0.168	0.540	−0.571	0.138	0.315	0.289
LA9WAT	0.759	0.292	0.180	−0.048	0.108	0.687	0.346	0.163	−0.157	0.113
NB9WAT	0.702	−0.385	0.017	0.148	−0.020	0.774	−0.306	0.076	−0.056	0.272
NN9WAT	0.652	−0.459	0.014	−0.002	−0.312	0.734	−0.434	0.069	−0.160	0.090
SG9WAT	0.682	0.231	0.060	−0.244	0.117	0.698	0.140	0.010	−0.405	0.117
NI9WAT	0.709	−0.452	0.012	−0.001	−0.204	0.796	−0.375	0.088	−0.048	0.048
DFA	−0.798	0.239	0.363	0.031	0.182	−0.753	0.214	0.263	−0.271	0.149
D50%A	−0.789	0.175	0.366	0.043	0.310	−0.717	0.126	0.242	−0.292	0.189
NFlPT	0.766	−0.236	0.416	0.203	0.007	0.764	−0.068	0.513	0.184	0.098
TNFlPP	0.256	−0.739	0.139	0.489	0.193	0.328	−0.577	0.399	0.559	0.111
NAFlPP	−0.564	0.243	0.242	0.109	0.233	−0.599	0.265	0.259	0.220	−0.278
FlL	0.760	0.268	0.438	−0.125	0.003	0.705	0.508	0.327	0.003	−0.118
FlW	0.714	0.140	0.540	−0.049	0.061	0.627	0.424	0.464	0.108	−0.153
SlL	0.761	0.317	0.376	−0.193	0.042	0.680	0.486	0.318	−0.077	−0.153
SlD	0.480	−0.184	0.303	−0.337	−0.172	0.117	0.274	−0.159	0.294	−0.325
OL	0.541	0.507	−0.317	0.277	−0.282	0.503	0.342	−0.366	−0.078	0.190
OD	0.458	0.570	−0.396	0.364	−0.100	0.140	0.638	−0.260	0.650	−0.178
OA	0.434	0.598	−0.229	0.477	−0.062	0.077	0.604	−0.236	0.667	−0.202
OP	0.496	0.562	−0.296	0.466	−0.012	0.148	0.634	−0.256	0.649	−0.187
SGD	0.653	0.429	−0.181	0.156	0.318	0.702	0.407	−0.215	−0.028	0.360
SGL	0.742	0.270	−0.134	0.210	0.335	0.792	0.261	−0.220	0.025	0.234
DFFE	−0.706	0.242	0.292	0.086	0.417	−0.685	0.241	0.319	0.149	0.256
D50%FS	−0.747	0.222	0.360	0.262	0.281	−0.727	0.227	0.395	0.163	0.301
DFFR	−0.725	0.204	0.317	0.217	0.365	−0.695	0.232	0.373	0.243	0.272
NFrPT	0.674	−0.231	0.480	0.284	0.106	0.671	−0.046	0.585	0.224	−0.009
TNFrPP	0.244	−0.746	0.119	0.499	0.195	0.321	−0.600	0.389	0.545	0.105
FWPP	0.628	0.148	0.593	−0.200	0.108	0.570	0.269	0.545	−0.265	−0.302
AFW	0.400	0.219	0.601	−0.415	−0.059	0.343	0.297	0.393	−0.498	−0.393
FrL	0.522	0.531	−0.178	−0.034	0.126	0.591	0.502	−0.306	−0.053	0.365
FrD	0.534	0.413	−0.409	−0.019	0.317	0.542	0.330	−0.509	0.070	0.376
NLPF	0.643	0.359	−0.296	−0.050	0.406	0.699	0.217	−0.299	0.034	0.222
1st FrSP	0.158	−0.497	−0.444	−0.322	0.456	0.281	−0.606	−0.341	−0.001	−0.298
50% FrSP	0.285	−0.481	−0.406	−0.401	0.344	0.398	−0.486	−0.342	0.049	−0.344
100% FrSP	0.389	−0.542	−0.321	−0.445	0.387	0.537	−0.512	−0.289	0.084	−0.310
FPT	0.378	0.354	−0.208	−0.521	0.147	0.617	0.116	−0.273	−0.449	−0.176
TFYPH	0.617	0.274	0.597	−0.044	−0.036	0.568	0.432	0.534	−0.190	−0.110
Eigen value	13.545	6.232	4.167	2.923	2.024	13.330	5.904	4.105	3.405	2.005
Percentage variance	36.609	16.844	11.262	7.899	5.470	36.028	15.956	11.093	9.202	5.420
Cum. var. proportion	36.609	53.453	64.715	72.614	78.083	36.028	51.984	63.077	72.280	77.699

Note

PH9WAT (cm), NL9WAT, LA9WAT (cm^2^), NB9WAT, NN9WAT, NI9WAT, and SG9WAT (cm) (Plant height; Number of leaves; Leaf area; Number of branches; Number of nodes; Number of internodes; and Stem girth at 9 weeks after transplanting, respectively).

100% FrSP, Number of days to 100% fruit spoilage; 1st FrSP, Number of days to first fruit spoilage; 50% FrSP, Number of days to 50% fruit spoilage; AFW (g), Average fruit weight; Cum. var., Cumulative variance; D50%A, Number of days to 50% anthesis; D50%FS, Number of days to 50% fruit set; DFA, Number of days to first anthesis; DFFE, Number of days to first fruit emergence; DFFR, Number of days to first fruit ripening; Dim, Dimension; FlL (cm), Flower length; FlW (cm), Flower width; FPT (cm), Fruit pericarp thickness; FrD (cm), Fruit diameter; FrL (cm), Fruit length; FWPP (g), Fruit weight per plant; NAFlPP, Number of aborted flowers per plant; NFlPT, Number of flowers per truss; NFrPT, Number of fruits per truss; NLPF, Number of locules per fruit; OA (cm^2^), Ovary area; OD (cm), Ovary diameter; OL (cm), Ovary length; OP (cm), Ovary perimeter; SGD (cm), Stigma diameter; SGL (cm), Stigma length; SlD (cm), Style diameter; SlL (cm), Style length; TFYPH (t/ha), Total fruit yield per hectare; TNFlPP, Total number of flowers per plant; TNFrPP, Total number of fruits per plant.

Scree plot describes the percentage of variance related to each component which is usually obtained through a graph drawn between cumulative percentage of explained variances or Eigen values or combination of both and principal components (Woods & Edwards, 2011). However, it is worthy of note of the fact an Eigen value is the proportion of variance explained by each component. The scree plots are shown in Figures 8 and 10 and they exhibited 10 principal components and indicated the highest variation in Dim 1, which explained maximum variation in both first and second season data set. Hence, accessions selection based on this particular principal component or dimension is important. Figures 7 and 9 show the traits associations for the first and second season evaluation, respectively, which were reviewed based only on biplot of first and second dimensions in each season planting. The review was such that the horizontal axis was associated with the first dimension, and the vertical axis was associated with the second dimension. The traits grouping and location on the quadrants were determined by the factor loadings of each trait on the two axes while the component value would be obtained by summing the values of the two dimensions for each season data set.

From the first and second dimensions, we could see that virtually all the traits appeared discriminating and contributed maximally toward the total variability present in the evaluated tomato accessions in both seasons. However, traits, namely number of flowers per truss, style length, flower length, leaf area, stigma length, flower width, number of internodes, number of branches, stem girth, number of fruits per truss, stigma diameter, number of nodes, plant height, number of locules per fruit, fruit weight per plant, total fruit yield per hectare, fruit pericarp thickness, ovary diameter, ovary perimeter, ovary area, fruit length, ovary length, and fruit diameter were outstandingly high based on the magnitude of their factor loadings. This suggests that these enlisted traits explained the majority of variations available in tomato and of course contributing to fruit yields. Therefore, to achieve genetic improvement in tomato, selection should be directed toward these traits. Some authors have reported similar results in the past mentioning some of the traits implicated in the present study. For instance, Kiran et al. (2017) reported plant height and number of fruits per plant as the major contributors toward genetic divergence available among the tomato accessions screened. These traits according to them were followed by average fruit weight, fruit yield per plant, number of locules per fruit, number of bunches per plant, total soluble solids, number of primary branches per plant, fruit length, number of fruits per bunch, and fruit girth in decreasing order of their contribution. Mahesh et al. (2006) and Singh et al. (2008) had earlier reported the importance of plant height, average fruit weight, and number of fruits per plant and fruit yield per plant to the genetic diversity observed in tomato. Hence, they suggested that such traits will provide a good scope for efficient tomato fruit yield improvement by direct selection to obtain worthy results. The findings of the present study are also in consonance with those of Sharma et al. (2011) and Rajeev and Reddy (2012).

Principal component/dimension biplots had been used by many researchers, such as Ahmadizadeh and Felenji (2011) in potato, Afuape et al. (2011) and Sethuraman et al. (2007) in sweet potato, and Rai et al. (2017) in tomato to select the best genotype based on trait association. According to Silva and Padovani (2006), normally, the first two dimensions account for the significance of a larger number in magnitude as contributing more to the total variation. Ullah et al. (2007) reported that usually, the first dimension is seen as the most essential as a result of its perceived greatest contribution to the phenotypic variation available among accessions. De C. Leite and de Oliveira (2015) in their report stated that the first two principal components explained 77.90% of the percentage cumulative variation in their work on disease severity and oil content of sunflower. However, in the present study, the first five principal components could explain 78.08% and 77.70% of percentage cumulative variance for the first and second seasons, respectively. It was observed that the first two components appeared in smaller magnitude of total variation with Eigen values higher than 2.0. In another report, Rai et al. (2017) stated that 95% of the total variation found among 56 tomato genotypes was accounted for by the first 10 dimensions. However, of the 14 total PCs which explained 100% total variability, the first five component axes at an Eigen value of 1.0 expressed cumulative variance of 76.64%. Furthermore, Rehman et al. (2019) reported 77.20% of total variation present among 25 tomato accessions at an Eigen value of 1.0, which was explained by the first six PCs. Evgenidis et al. (2011) also noted that PC1 and PC2 accounted for a cumulative proportion of variability of 78.77% in tomato hybrids and inferred that the most important characters for the separation are those with the highest magnitude of factor loading on the first two dimensions. Akinwale et al. (2014) noted with concern in their review article that no study has been able to determine when the total percentage of variation accounted for by a biplot should be considered too small or insignificant to give a reasonable judgment. According to them, nevertheless, general assumptions have projected cumulative proportion of variation less than 40% with higher Eigen values probably greater than 1.0 or 2.0 as being too small to make inference. Going by this argument, from the present study, the first component produced total variation less than 40% with higher Eigen values in both seasons, and as a result, was not enough to make conclusion, hence, the need to consider other dimensions. Pradhan et al. (2011) reported that Eigen values are derivatives of principal components, which are used to specify the relative discriminative power of the axes and their associated traits.

### 3.3 Hierarchical cluster analysis

Cluster analysis is used for the identification of different clusters based on the grouping patterns of the accessions evaluated (Nankar et al., 2020). It has demonstrated effective classification of genetic materials which of course is significantly helpful in conserving their biodiversity, and, hence, utilization in crop improvement program (Shukla et al., 2010).

**FIGURE 11:**
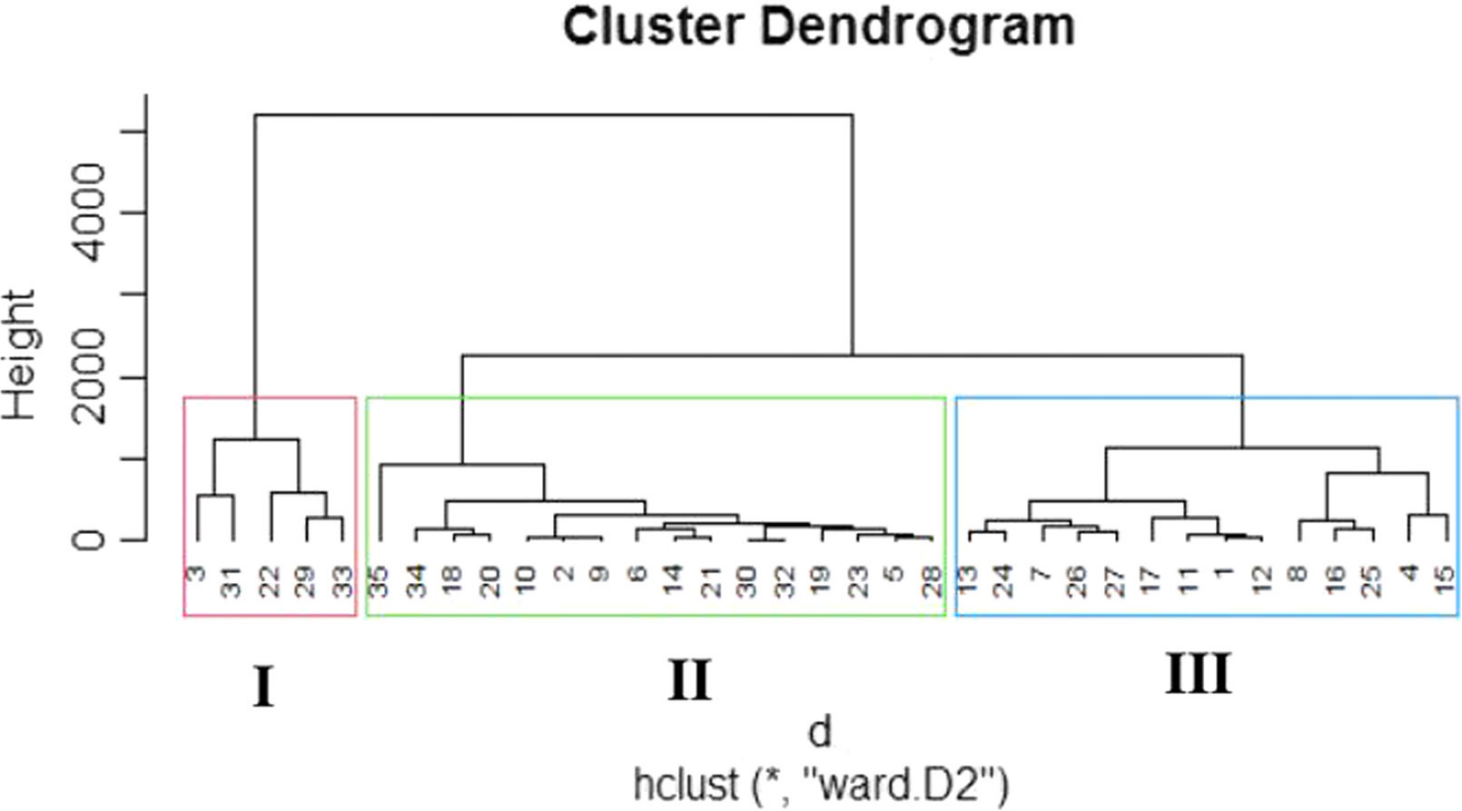
Hierarchical cluster dendrogram for tomato 1st season characterization

**FIGURE 12:**
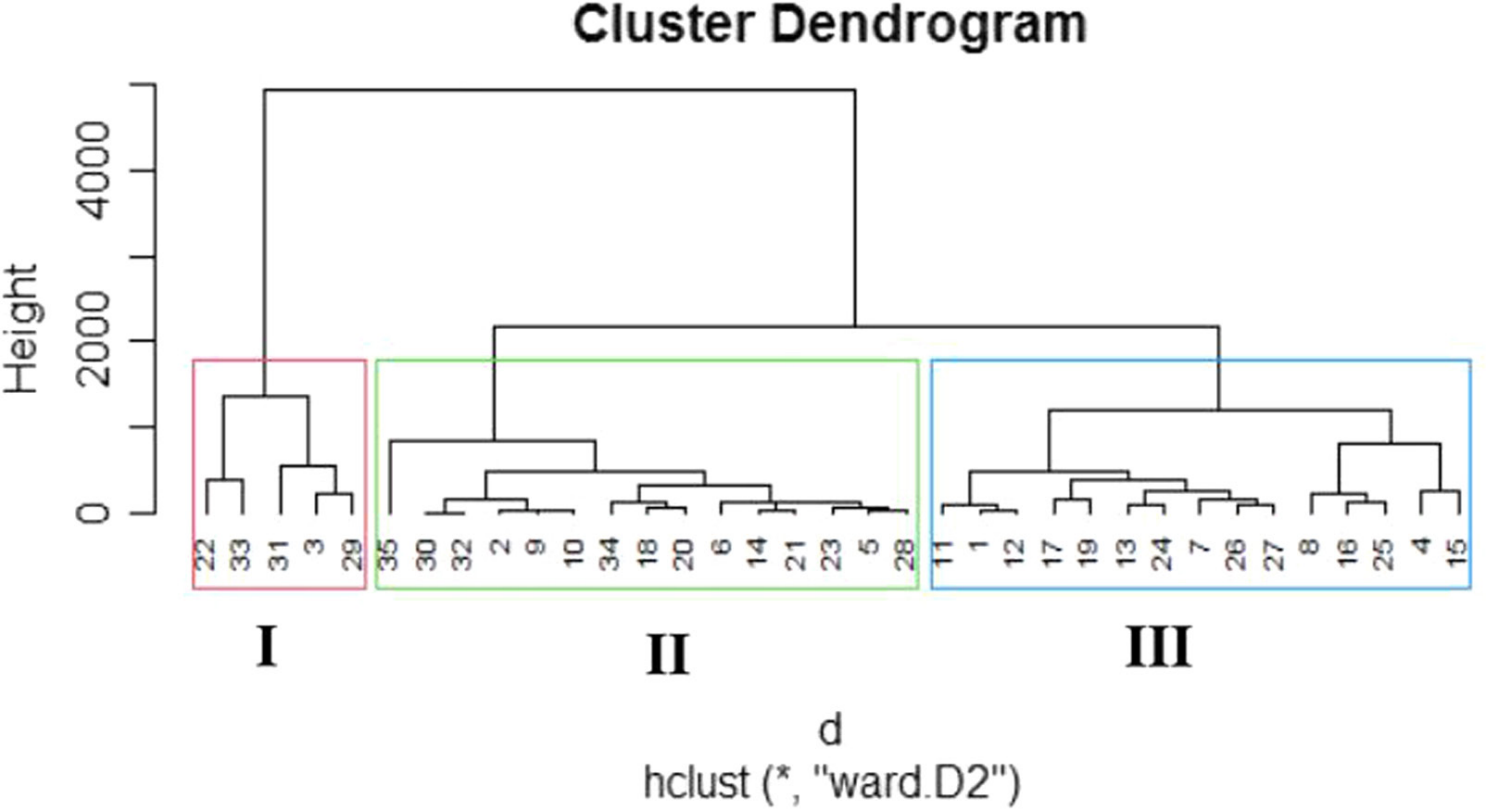
Hierarchical cluster dendrogram for tomato 2nd season characterization

According to the dendrogram, cluster analysis grouped 35 tomato accessions in both the first and second evaluations into three clusters as shown in Figures 11 and 12. In the first experiment, cluster I comprised of five accessions followed by 16 and 14 accessions, respectively, in clusters II and III, whereas in the second evaluation clusters II and III shared 15 accessions each leaving cluster I with 5 accessions as in the first planting. The first evaluation showed that cluster I comprised of ‘CLN2498D’, ‘PT4722A’, ‘Ruma’, ‘Tropimech’, and ‘UC Dan India’; cluster II included ‘CLN2468B’, ‘CLN2545B’, ‘CLN2585C’, ‘CLN2714G’, ‘CLN2714H’, ‘CLN2777E’, ‘Dan Holland’, ‘Darika’, ‘Gadar’, ‘Melka Salsa’, ‘PT4722B’, ‘Rukuta’, ‘Tima’, ‘UC 82 B’, ‘UCT’, and ‘Wild parent’, while cluster III consisted of ‘CLN2116B’, ‘CLN2498F’, ‘CLN2585D’, ‘CLN2679F’, ‘CLN2762A’, ‘CLN2768A’, ‘CLN2777A’, ‘CLN2777F’, ‘CLN2777G’, ‘CLN2777H’, ‘Rio Grande’, ‘Roma Savanna’, ‘Roma VF’ [Ethio.], and ‘Roma VF’ [Nig.]. Similar trend was followed in the second tomato screening, except that ‘Darika’ got drifted into cluster III from cluster II. The same similarity axis was maintained in both evaluation seasons. The comparison between the population means from ANOVA and accession cluster mean values is shown in Tables 2 and 3. For the first planting season, cluster I consistently and maximally showed better performance in virtually all the quantitative traits studied including the related growth, floral, and fruit traits compared to clusters II and III as well as the population means. However, minimum differences were observed between clusters II and III, and the population means in most of the traits studied due to lower value of genetic diversity. Cluster III could only take up leadership for fruit length. Clusters I and III gave values which were higher than the population means for number of leaves, number of branches, number of nodes, number of internodes, stem girth, number of flowers per truss, flower length, flower width, style length, ovary length, ovary perimeter, number of fruits per truss, fruit weight per plant, average fruit weight, fruit length, and total fruit yield. The same cluster groups indicated the least number of days to observe their reproductive functions which was also lower than those recorded for population means. On the contrary, cluster II constantly showed values lower than both clusters I and III as well as the population means for all the traits mentioned and took longer days to complete their reproductive processes. Total number of flowers per plant, number of aborted flowers per plant, total number of fruits per plant, and fruit diameter projected clusters I and II as having performed higher than the population means. The same result was applicable with regards to traits that expressed tomato shelf‐life performance, such as number of days to first, 50%, and 100% fruit spoilage, as clusters I and II took leadership in those traits as well. In the second season of tomato evaluation (Table 3), the cluster means followed the similar pattern as in the first trial except that cluster III had values which were higher than the population means. However, cluster III was still lower than those of cluster I for plant height and fruit pericarp thickness. Cluster II showed a decline, slightly lower than the population mean for fruit diameter unlike the result obtained in the first outing, although it was also with slight increase higher than the population mean.

**TABLE 2:** Cluster means of growth, floral, and fruit traits of the first season characterization of tomato accessions

Growth Traits	Cluster A	Cluster B	Cluster C	Pop. mean	Floral Traits	Cluster A	Cluster B	Cluster C	Pop. mean	Fruit Traits	Cluster A	Cluster B	Cluster C	Pop. mean
PH9WAT	95.45	69.15	73.00	74.45	DFA	20.72	23.99	22.53	22.94	DFFE	28.68	31.49	29.52	30.30
NL9WAT	65.20	57.74	61.97	60.50	D50%A	24.13	27.33	25.19	26.02	D50%FS	36.07	39.27	38.52	38.68
LA9WAT	594.67	253.60	332.80	334.00	NFlPT	9.88	6.40	8.64	7.79	DFFR	55.87	59.77	58.67	59.00
NB9WAT	10.53	6.56	9.67	8.37	TNFlPP	83.46	70.25	61.56	68.66	NFrPT	10.25	5.66	7.83	7.18
NN9WAT	31.15	20.90	28.58	25.43	NAFlPP	10.26	10.90	9.05	10.07	TNFrPP	77.22	58.45	48.15	57.01
NI9WAT	18.17	11.93	14.71	13.93	FlL	0.74	0.45	0.60	0.55	FWPP	2360.09	367.57	939.66	881.06
SG9WAT	9.73	5.87	6.58	6.71	FlW	0.27	0.13	0.20	0.18	AFW	32.68	11.94	20.52	18.33
					SlL	0.70	0.35	0.50	0.46	FrL	7.89	7.14	8.02	7.60
					SlD	0.04	0.03	0.04	0.04	FrD	4.64	4.43	4.35	4.42
					OL	0.14	0.12	0.15	0.13	NLPF	4.55	3.55	3.62	3.72
					OD	0.14	0.12	0.13	0.13	1st FrSP	12.09	11.46	8.91	10.53
					OA	0.02	0.01	0.01	0.01	50% FrSP	19.77	18.82	17.03	18.24
					OP	0.41	0.32	0.37	0.35	100% FrSP	28.76	27.10	24.75	26.40
					SgL	0.05	0.02	0.03	0.03	FPT	5.33	4.57	4.48	4.64
					SgD	0.10	0.05	0.06	0.06	TFY	62.96	20.87	46.81	37.26

Note

**Growth traits:** PH9WAT (cm), NL9WAT, LA9WAT (cm^2^), NB9WAT, NN9WAT, NI9WAT, and SG9WAT (cm) (Plant height; Number of leaves; Leaf area; Number of branches; Number of nodes; Number of internodes; and Stem girth at 9 weeks after transplanting, respectively).

**Floral traits:** D50%A, Number of days to 50% anthesis; DFA, Number of days to first anthesis; FlL (cm), Flower length; FlW (cm), Flower width; NAFlPP, Number of aborted flowers per plant; NFlPT, Number of flowers per truss; OA (cm^2^), Ovary area; OD (cm), Ovary diameter; OL (cm), Ovary length; OP (cm), Ovary perimeter; SgD (cm), Stigma diameter; SgL (cm), Stigma length; SlD (cm), Style diameter; SlL (cm), Style length; TNFlPP, Total number of flowers per plant.

**Fruit traits:** 100% FrSP, Number of days to 100% fruit spoilage; 1st FrSP, Number of days to first fruit spoilage; 50% FrSP, Number of days to 50% fruit spoilage; AFW (g), Average fruit weight; D50%FS, Number of days to 50% fruit set; DFFE, Number of days to first fruit emergence; DFFR, Number of days to first fruit ripening; FPT (cm), Fruit pericarp thickness; FrD (cm), Fruit diameter; FrL (cm), Fruit length; FWPP (g), Fruit weight per plant; NFrPT, Number of fruits per truss; NLPF, Number of locules per fruit; Pop., Population; TFYPH (t/ha), Total fruit yield per hectare; TNFrPP, Total number of fruits per plant.

**TABLE 3:** Cluster means of growth, floral, and fruit traits of the second season characterization of tomato accessions

Growth Traits	Cluster A	Cluster B	Cluster C	Pop. mean	Floral Traits	Cluster A	Cluster B	Cluster C	Pop. mean	Fruit Traits	Cluster A	Cluster B	Cluster C	Pop. mean
PH9WAT	98.89	71.19	77.95	77.80	DFA	21.40	23.71	23.36	23.28	DFFE	28.00	29.76	27.52	28.72
NL9WAT	66.82	60.10	66.76	63.92	D50%A	24.97	27.07	26.51	26.60	D50%FS	35.17	37.77	36.25	36.91
LA9WAT	592.65	258.35	336.63	336.18	NFlPT	9.92	6.44	8.62	7.81	DFFR	55.20	57.80	56.44	57.23
NB9WAT	10.81	6.56	9.94	8.66	TNFlPP	81.52	66.45	59.13	65.19	NFrPT	10.08	5.48	7.67	7.06
NN9WAT	31.08	20.65	28.20	25.08	NAFlPP	10.36	10.54	8.79	9.85	TNFrPP	76.17	54.41	46.10	53.67
NI9WAT	18.35	11.67	14.45	13.74	FlL	0.78	0.48	0.66	0.59	FWPP	2,260.52	355.69	929.64	862.21
SG9WAT	9.84	6.19	6.98	6.96	FlW	0.30	0.16	0.24	0.22	AFW	32.12	12.51	21.48	18.96
					SlL	0.71	0.39	0.58	0.51	FrL	8.10	7.18	8.28	7.78
					SlD	0.06	0.06	0.06	0.06	FrD	4.78	4.55	4.57	4.62
					OL	0.15	0.14	0.17	0.15	NLPF	4.78	3.75	4.01	4.02
					OD	0.18	0.16	0.17	0.17	1st FrSP	12.45	11.87	9.48	10.87
					OA	0.03	0.02	0.02	0.03	50% FrSP	20.84	19.30	17.56	18.67
					OP	0.58	0.50	0.52	0.54	100% FrSP	29.41	27.35	25.24	26.75
					SgL	0.07	0.04	0.05	0.05	FPT	5.50	4.46	4.79	4.76
					SgD	0.11	0.06	0.08	0.08	TFY	62.68	19.25	46.58	37.06

Note

**Growth traits:** PH9WAT (cm), NL9WAT, LA9WAT (cm^2^), NB9WAT, NN9WAT, NI9WAT, and SG9WAT (cm) (Plant height; Number of leaves; Leaf area; Number of branches; Number of nodes; Number of internodes; Stem girth at 9 weeks after transplanting, respectively).

**Floral traits:** D50%A, Number of days to 50% anthesis; DFA, Number of days to first anthesis; FlL (cm), Flower length; FlW (cm), Flower width; NAFlPP, Number of aborted flowers per plant; NFlPT, Number of flowers per truss; OA (cm^2^), Ovary area; OD (cm), Ovary diameter; OL (cm), Ovary length; OP (cm), Ovary perimeter; SgD (cm), Stigma diameter; SgL (cm), Stigma length; SlD (cm), Style diameter; SlL (cm), Style length; TNFlPP, Total number of flowers per plant.

**Fruit traits:** 100% FrSP, Number of days to 100% fruit spoilage; 1st FrSP, Number of days to first fruit spoilage; 50% FrSP, Number of days to 50% fruit spoilage; AFW (g), Average fruit weight; D50%FS, Number of days to 50% fruit set; DFFE, Number of days to first fruit emergence; DFFR, Number of days to first fruit ripening; FPT (cm), Fruit pericarp thickness; FrD (cm), Fruit diameter; FrL (cm), Fruit length; FWPP (g), Fruit weight per plant; NFrPT, Number of fruits per truss; NLPF, Number of locules per fruit; Pop., Population; TFYPH (t/ha), Total fruit yield per hectare; TNFrPP, Total number of fruits per plant.

The cluster analysis including the means gave an indication that the tomato accessions were classified based on their related vegetative, floral, and fruit traits performance. In the measured related growth, floral, and fruit traits, cluster II consistently performed poorly. It had prolonged vegetative and reproductive phase compared to clusters I and III. Cluster I showed higher morphological traits and fruit yield performance over clusters II and III including the population means. This suggests that this particular cluster expressed the best agronomic characteristics and yield potentials and would respond more to selection than the other clusters assuming fruit yield is the target. This corroborates with the opinion of Staub et al. (2005) in cucumber. The grouping pattern of the tomato accessions did not obey their source or geographical distribution as they appeared randomly in the clusters. This suggests that accessions of similar source distribution which fell into different clades heedless of origin show a sign of broad genetic base of the accessions. This could possibly have been because the genetic materials used for the evaluation were not products of selection but germplasm collation from different sources, with a self‐pollinating crop known for its narrow genetic base. Schouten et al. (2019) and Vargas et al. (2020) reported that breeding has increased the diversity of cultivated tomato, especially with its related wild relatives both at the genotypic and phenotypic levels. Different authors such as Osawaru et al. (2013) in West African okra (*Abelmoschus caillei* [A. Chev.] Stevels), Prasad et al. (2001) and Ene et al. (2016b) in cucumber, and Nankar et al. (2020) in tomato have reported the same result. The superior related vegetative, floral, and fruit traits performance of the accessions aligned in cluster I over clusters II and III in both seasons indicates gainful exploitation in tomato improvement programs. From the present investigation, it could be stated that cluster analysis obviously can be considered as an effective tool to assort tomato accessions based on their performance relatedness for the traits studied. Feng‐Mei et al. (2006) and Iqbal et al. (2014) reported cluster analysis as having provided authentic foundation for selection of base materials to outline future improvement plans in tomato. Nevertheless, the authors in addition mentioned that while the selection of base material is being made, genetic barriers must be handled, as well as choosing appropriate breeding methods to obtain anticipated genetic improvements for traits desired. Cluster analysis had been utilized extensively in tomato germplasm improvement studies on different quantitative and qualitative traits in various parts of the world (Iqbal et al., 2014; Kiran et al., 2017; Mishra et al., 2018; Nankar et al., 2020; Prakash & Vijay, 2017; Rehman et al., 2019).

## 4 CONCLUSIONS

Multivariate analysis is an efficient technique to quantify diversity among germplasm due to trait variability. Generally, multivariate analysis gave perception of tomato accessions separation into different groups. From the present study, GT biplot projected ‘wild parent’, ‘CLN2498D’, ‘CLN2498F’, ‘UC Dan India’, ‘Ruma’, ‘PT4722A’, ‘CLN2679F’, ‘CLN2585C’, and ‘CLN2585D’ as best performers for most of the related growth, floral, and fruit traits including fruit production earliness in the two seasons; whereas ‘UC 82 B’, ‘Tima’, ‘CLN2714H’, and ‘CLN2714G’ consistently maintained poor performance for most of the agronomic traits examined including total fruit yield and they took longer period to express their phenological traits indicating poor gene makeup. Days to floral and fruit appearance as well as fruit maturity are essential component in tomato production. This is because it is a transition for the initiation of reproductive stage in the lifecycle of the plant followed by actual productive harvest.

The principal component analysis in both seasons revealed that selection for number of flowers per truss, style length, flower length, leaf area, stigma length, flower width, number of internodes, number of branches, stem girth, number of fruits per truss, stigma diameter, number of nodes, plant height, number of locules per fruit, fruit weight per plant, total fruit yield per hectare, fruit pericarp thickness, ovary diameter, ovary perimeter, ovary area, fruit length, ovary length, and fruit diameter will increase tomato fruit yield. These traits should thus be given priority during selection process targeted at tomato fruit yield improvement.

Tomato accessions clustering into different classes indicate comparatively high genetic variation among accessions. Results suggest adequate genetic variability in the studied accessions to support selection in both planting seasons. Due to the observed diversity based on cluster mean performance between clusters I and II and (or) clusters I and III, it could be concluded that accessions of these clusters are complementary for maximum traits performances and could be chosen for planned hybridization to develop promising F_1_ hybrids with good heterotic effect or transgressive segregants in subsequent generations.

## CONFLICT OF INTEREST

The authors declare that there are no conflict of interest.
